# Racial Disparity and Triple-Negative Breast Cancer in African-American Women: A Multifaceted Affair between Obesity, Biology, and Socioeconomic Determinants

**DOI:** 10.3390/cancers10120514

**Published:** 2018-12-14

**Authors:** Sumit Siddharth, Dipali Sharma

**Affiliations:** Department of Oncology, Johns Hopkins University School of Medicine and the Sidney Kimmel Comprehensive Cancer Center at Johns Hopkins, Baltimore, MD 21231, USA

**Keywords:** triple negative breast cancer, racial disparity, African-American, risk factors, obesity

## Abstract

Triple negative breast cancer (TNBC) is a molecularly heterogeneous disease whose incidence is disproportionately higher in African American (AA) women compared to European American (EA) women. Earlier onset, more advanced stage at diagnosis, and aggressive tumor phenotype are some of the characteristic features of TNBC in women with African ethnicity in comparison to EA women, denoting one of the most significant examples of racial disparity in oncology. It is still contentious whether health disparities result in aggressive behavior of TNBC in AA women or it is indeed a molecularly distinct disease. Given the “gaps-in-knowledge” surrounding racial disparity in TNBC, this review discusses various socioeconomic factors and the genetic predispositions contributing to poor prognosis of TNBC in AA women. While socioeconomic factors may contribute to poorer survival, multiple preclinical and clinical studies suggest inherent genetic risk factors and aberrant activation of oncogenic pathways in AA TNBC. Additionally, AA women are more likely to be obese and obesity is known to drive a molecular circuitry resulting in aggressive tumor progression indicating a potential obesity-TNBC axis at work in AA women. Given the multifactorial nature of AA TNBC, a transdisciplinary approach may help bridge the disparity that exists between AA and EA TNBC.

## 1. Introduction

**Figure 1:**
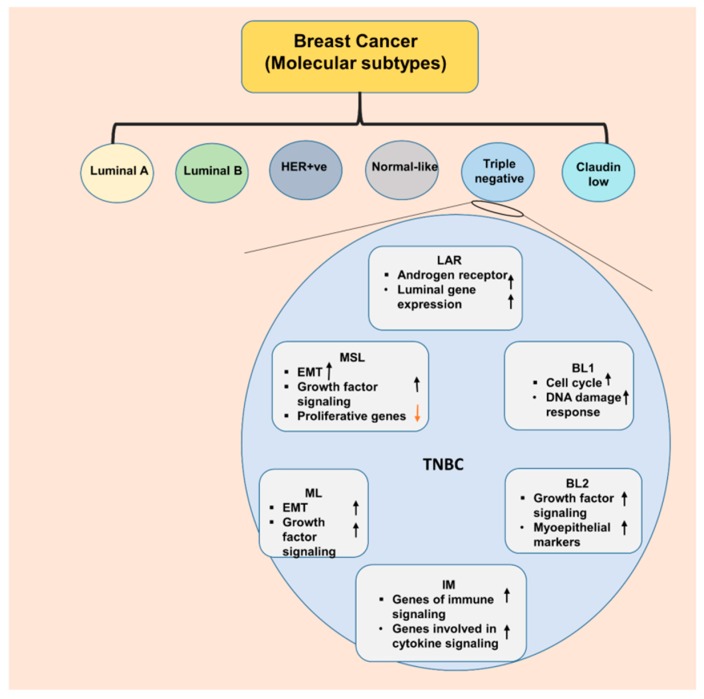
Schematic illustrating the molecular subtypes of breast cancer. The blue circle groups six different subtypes of triple-negative breast cancer (TNBC) and demonstrate the characteristics of these subtypes. Subtypes of TNBC: BL1, basal-like 1; BL2, basal-like 2; IM, immunomodulatory; ML, mesenchymal-like; MSL, mesenchymal stem-like; LAR, luminal androgen receptor.

Breast cancer is the second-most common cancer and a leading cause of cancer-related mortality for women in United States [1]. Approximately 266,120 new invasive breast cancer cases are estimated in 2018 according to American Cancer Society. Currently, there are more than 3.1 million breast cancer survivors in United States [2]. Breast cancer has been historically sub-classified on the basis of three molecular markers; estrogen receptor (ER), progesterone receptor (PR) and human epidermal growth factor receptor 2 (EGFR2/Her2), whose presence/absence guide the prognosis and therapeutic interventions. ER/PR positive breast cancer are candidates for endocrine therapy regimens and Her2 positive breast cancer can be targeted with anti-Her2 therapies whereas ER/PR/Her2 negative or triple-negative breast cancer (TNBC) lack any targeted therapies [3]. Owing to technical advances, breast cancer is now classified on the basis of immunohistochemical markers and complementary DNA microarrays [4,5] into six different subtypes, namely, luminal A (ER+ and/or PR+ and HER2−), luminal B (ER+ and/or PR+ and HER2+), HER2 overexpressing (ER−, PR− and HER2+), normal-like, claudin-low and basal-like/TNBC (ER/PR/HER2 negative, cytokeratin 5/6+ and/or epidermal growth factor receptor+) (Figure 1) [3,5,6]. This classification varies in prognosis and therapeutic targets but corresponds to the histopathologic features considering different types of breast epithelial cells; luminal and basal, which can be immunohistochemically distinguished based on the expression of keratins. Keratins 8/18 are expressed by the luminal cells while keratins 5/6 and 17 are expressed by the basal cells [7,8]. High cost of molecular analysis along with limited availability of bioinformatics expertise and the infrastructure are some of the limitations that have prevented the introduction of this classification system as standard practice into clinics.

## 2. Triple Negative Breast Cancer—an Aggressive Subtype

**Figure 2:**
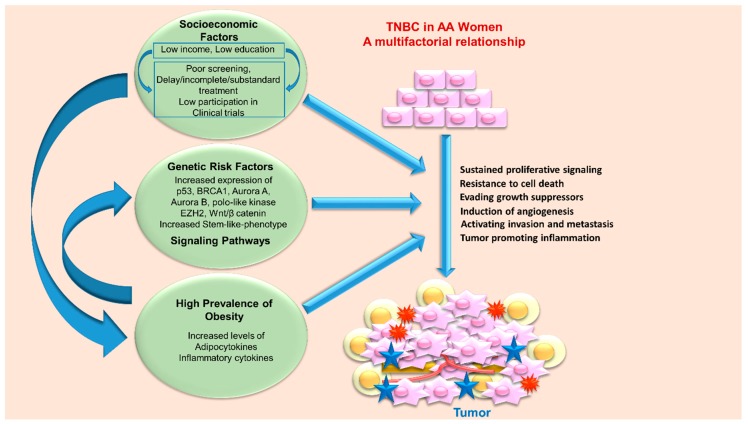
Overview of various socioeconomic and biological factors contributing to disparity in TNBC progression in African American (AA) versus European American (EA) women. Various socioeconomic factors such as low income and poor access to health care can aid in high prevalence of obesity. Obese state can modify various signaling pathways and directly impact various tumor-promoting biological process including growth, invasion, and migration. These socioeconomic and biological factors contribute to TNBC progression in AA women directly or indirectly.

Triple negative breast cancer (TNBC) represents a class of heterogeneous cancer cells that exhibit different biological features, clinical presentations, therapeutic response and outcomes. TNBC encompasses 15% to 20% of all breast cancers [9]. TNBC are associated with worse prognosis, early relapse after standard chemotherapy, a high frequency of metastasis to lung, liver and brain and a low overall survival compared to other breast cancer subtypes [10]. To date most clinical trials utilize overall survival (OS) and progression-free survival (PFS) as the endpoints to evaluate the efficacy of therapeutic interventions but variability in postprogression survival (PPS) negatively impacts the correlation between OS and PFS and confounds the conclusions. A recent study including 472 patients evaluated the suitability of OS, PFS, and PPS as different measures of outcomes and concluded that clinical trials should adopt different endpoints based on the tumor characteristics to evaluate the benefits of new drugs. Importantly, OS can be a better endpoint for TNBC patients where PPS is low in contrast to luminal A/B and Her2+ tumors where the PPS is generally longer than a year [11]. Based on molecular profiling, Lehmann and colleagues showed that TNBC can be subdivided into 6 subgroups: basal-like 1 (BL1), basal-like 2 (BL2), immunomodulatory (IM), mesenchymal-like (M), mesenchymal stem-like (MSL), and luminal androgen-receptor (LAR) expressing; and have differential sensitivity to therapeutic agents (Figure 1) [12]. BL1 and BL2 are most sensitive to cisplatin, mesenchymal subgroup responds to dasatinib (SRC inhibitor) and NVP-BEZ235 (PI3K/mTOR inhibitor) while LAR are amenable to bicalutamide (an antiandrogen) and alvespimycin (an HSP90 inhibitor) therapy [12]. Basal-like breast cancer, characterized by the presence of genes normally found in basal or myoepithelial cells of the normal breast [5] came into existence owing to the microarray-based expression profiling studies [5]. While TNBC and basal-like breast cancers (BLBC) have multiple overlapping features and these terms are often used interchangeably, both have some important unique features thus have to be acknowledged as distinct subtypes of TNBC [13]. Basal-like tumors possess higher TP53 mutations (44% versus 15%, *p* = 0.001), complex mitotic index (odds ratio = 11.0; 95% confidence interval 5.6–21.7), greater nuclear pleomorphism (odds ratio = 9.7; 95% confidence interval 5.3–18.0) and higher combined grade (odds ratio = 8.3; 95% confidence interval 4.4–15.6). TNBC also encompass other breast cancer subtypes like claudin-low tumors which contain cells with stem cell properties and epithelial to mesenchymal transition potential, interferon rich subgroup encompassing tumors with a significantly better prognosis over other TNBCs [14,15]. Epidemiological studies have reported that TNBC are more common in women of African ancestry in comparison to other ethnic groups [16,17] and TNBC in African American (AA) women is associated with worse clinical outcomes compared to TNBC in European American (EA) women [10]. The age adjusted cumulative incidence rate of all subtypes of breast cancer is slightly lower in AA women in comparison to EA women with 124.3/100,000 cases in AA versus 128.1/100,000 cases in EA in United States; but AA women exhibit a 42% higher mortality rate than EA women [18]. Epidemiological, clinical and preclinical evidence reveal that the contributory factors for such a disparity encompass both biological and socio-economic causes (Figure 2) [19]. A study comparing breast cancer among AA, EA and Hispanic women reported that AA tumors were more likely to be associated with worse pathological characteristics such as larger tumors with less differentiated cancer cells [19].

## 3. Triple Negative Breast Cancer—Higher Prevalence in African American Women

**Figure 3:**
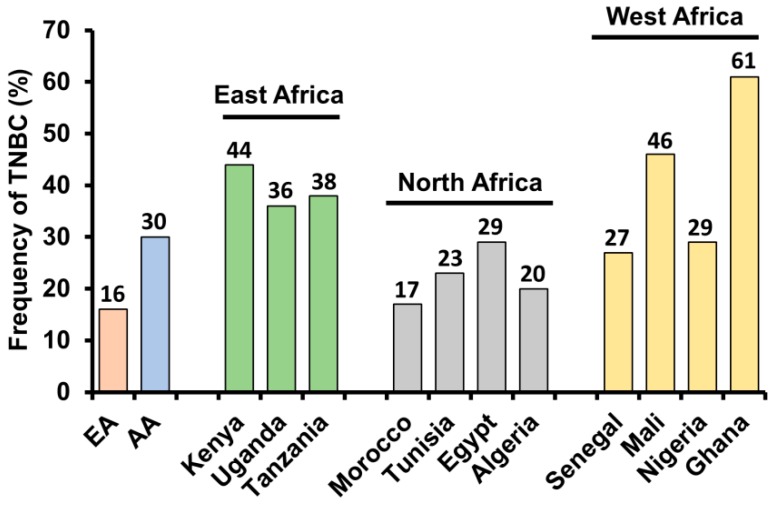
Prevalence of triple negative breast cancer (TNBC) is shown among European American (EA), African American (AA) and women with African ancestry.

Surveillance, Epidemiology, and End Results (SEER) data of women diagnosed with breast cancer revealed that TNBC incidences were higher in AA women than any other ethnic or racial group of all ages (*p* < 0.05) [20] irrespective of the fact that TNBC frequency itself varied across regional population of women of African ancestry [21]. TNBC is the most predominant cancer in sub-Saharan Africa (Figure 3) including 22 countries of Americas and the Caribbean [6,17,22,23]. The study by Huo and colleagues evaluating the distribution of molecular subtypes of invasive breast tumors in women (mean age 44.8 years) from various geographical areas of Nigeria and Senegal (507 women) found that basal-like TNBC was the most predominant cancer in this region [24]. Similarly, 46% of tumors were found to be triple negative in Bamako University Hospital in Mali where mean age of patients was 46 years [25]. Another case study of 1216 breast cancer patients from Soweto, South Africa revealed that 90% of women with breast cancer were black and showed 20% prevalence of TNBC which was consistent with the reported frequency of TNBC in AA women [26].

Bowen and colleagues interrogated a UK based breast cancer cohort and found that 22% of black women had TNBC in comparison to 15% of white women [27]. In a population-based study of North Carolina Breast Cancer Cohort of 878 AA women and 187 EA women breast cancer cases, it was observed that premenopausal AA women had higher basal breast cancer rate (39%) than postmenopausal AA (14%) or white women of similar age group (16%) [17]. Using the data available on tumor grade, stage, ER/PR/HER2 status, patient age and BMI index along with self-identified racial group, Stead and colleagues concluded that the probability of TNBC incidence in AA women was 3 fold higher in comparison to EA women irrespective of age (31% before 50 years versus 29% after 50 years) and obesity (29% obese versus 31% non-obese) [28]. To investigate the potential differences between TNBCs and other breast cancer sub-types pertaining to age, race, grade, diagnosis stage, socioeconomic status, and relative survival, Bauer et al. followed women diagnosed with TNBC from 1999 to 2003 using the population-based California Cancer Registry data. This study compared a total of 6370 TNBC cases to 44,704 cases diagnosed with other breast cancers and reported that the non-Hispanic black or black women under the age of 40 were more likely to have TNBC [16]. Examining 375,761 invasive breast cancers (including 276,938 non-Hispanic white and 21,681 non-Hispanic black) [29], Amirikia and colleagues found that both Hispanic (median age 54) and non-Hispanic (median age 57) black women were younger than non-Hispanic white women (median age 64) at the time of cancer incidence and had higher TNBC incidences in all age categories. Also, non-Hispanic black women younger than 44 years had the highest lifetime TNBC incidence rates and higher incidence rates of stage III and IV disease [29]. Examining data from the Yale TNBC cohort (50 AA and 86 EA tumors), Lindner et al, showed that basal subtype is more common in AA women [30]. Similar conclusions were put forth by Keenan et al. after analyzing TNBC cases from The Cancer Genome Atlas (TCGA) [31]. Evaluation of TCGA data also showed that TNBC (33.3% vs. 14.9%) and basal (34.8% vs. 16.1%) subtype is more prevalent in AA women in comparison to EA women [32]. The available epidemiological data indicates that although TNBC is not restricted to a specific age or ethnic group, its frequency is higher in women of African ancestry and is associated with survival disadvantage. Overall and recurrence-free survival varies greatly according to the breast cancer subtype with shortest survival associated with basal-like sub-type and HER2/ER negative breast cancer [17]. Since basal like/TNBC incidence is greater in AA women, it seems intuitive that AA women exhibit overall poor prognosis. Indeed, the poorest survival was observed in the non-Hispanic black women with only 14% 5-year relative survival [16]. These disparities suggest the presence/absence of oncogenes/tumor suppressor genes, mutations and altered signaling pathways that might predispose premenopausal AA women to TNBC. Along with molecular alterations, socioeconomic factors may also contribute to overall poor prognosis.

## 4. Triple Negative Breast Cancer Disparity in African American Women—Biology and Environment

### 4.1. Genetic Risk Factors

Compared to TNBC in EA women, TNBC in AA women show enhanced p53, BRCA1, Aurora A, Aurora B and polo like signaling networks [33]. BRCA1 mutant breast cancers are typically TNBC [34] and though TNBC incidences are higher in AA women compared to other ethnic groups, incidence of germline BRCA1 mutation is higher in European women. A study focusing on 155 high-risk families at University of Chicago, Illinois, USA, showed that AA women had lower pathogenic variant BRCA1 than non-Hispanic and non-Jewish Caucasians but had higher rate of sequence variations [35]. More importantly, these sequence variations that block BRCA1 function occur in AA women at a lower rate than in European women [36]. Enhancer of zeste homologue 2 (EZH2), a member of Polycomb group family, blocks BRCA1 function by inducing protein kinase B (PKB or AKT) dependent genomic instability [37]. Recent studies have linked EZH2 and aggressive TNBC in AA women. Overexpression of nuclear EZH2 significantly associated with basal-like TNBC in women of African descent in a joint study by Kleer and Newman including 100 invasive breast cancer cases in Ghanaian women [38]. A similar study encompassing 295 breast cancer patients from Netherlands also indicated a high EZH2 expression in basal-like TNBC. Interestingly, some epidemiological studies indicate a prominent role of EZH2 irrespective of ethnic origins [39]. Stewart et al. performed a broad differential gene expression analysis containing sub-type and stage-specific analysis of the breast cancer data from TCGA and found an almost 2 fold elevated expression of Aurora B [33]. There are multiple founder mutations in BRCA genes and genetic variations in other genes associated with breast cancer but their functional significance in TNBC incidence and progression in AA women is currently unclear.

### 4.2. Socioeconomic Factors

Multiple socioeconomic factors influence the access to standard care, novel treatments and inclusion in clinical trials and contribute to overall prognosis. AA women with breast cancer do not usually avail or have access to appropriate guideline-concordant therapeutic regimens including locoregional treatment, adjuvant radiotherapy, adjuvant systemic therapy and breast reconstruction. Lack of timely screening might result in delayed diagnosis and bigger tumor burden at diagnosis. In addition, limited access to standard treatment modalities might lead to increased tumor progression and poor overall survival. Another layer of complexity is that AA women with breast cancer generally have lower participation in clinical trials investigating novel drug combinations than their EA counterparts [40], which might result in lack of race-specific data for evaluating the efficacy of new drugs and unavailability of new treatment regimens. Owens et al., conducted an interesting study to assess the “awareness” and “confidence in clinical studies” among African-American people and evaluated their impact on overall participation in clinical trials pertaining to cancer research. They found that AA men and women not only had insufficient knowledge regarding clinical trials and informed consent process, they also lack confidence and trust in cancer research [41]. The findings of this study also state that AA men correlate clinical trials to the term “guinea pigs” and recognized clinical trials as experiments. Two factors—fear and mistrust, appeared as the most important obstacles for AA people to participate in clinical trials. Fear was usually associated with the harms caused during the clinical trial procedure and mistrust arose due to multiple historic unethical research studies for example, Tuskegee Syphilis Study conducted on AA people. Together, fear and mistrust regarding scientific community leads to non-participation of AA people in clinical trials resulting in lack of sufficient race-specific data [41]. A similar study was conducted by Haynes-Maslow et al. for the better understanding of the barriers responsible for low enrollment of African-American women in clinical trials. This study quotes an African-American woman stating, “It is important because we as a race are automatically standoffish and so afraid from past things. We all know about the Tuskegee and we just don’t feel comfortable participating in things, and it’s for lack of knowledge” [42].

Some other major contributors to treatment-related disparities include higher probability that AA breast cancer patients access under-resourced hospitals and have imperfect communication with health care providers as well as biased practices in the health care system. Meta-analysis of socioeconomic status adjusted breast cancer survival in >14,000 AA patients compared to >76,000 EA patients demonstrated a ~30% higher mortality hazard among AA patients [43]. Comorbidities such as diabetes and hypertension are also prominent factors responsible for survival differences among AA and EA breast cancer patients [44]. According to Hershman et al., insufficient delivery of adjuvant breast cancer chemotherapy leads to neutropenia in AA patients [45]. Interestingly, the Southwest Oncology group adjuvant therapy trial’s pooled analyses revealed persistent survival disadvantage for AA patients having breast and prostate cancer (hormonally driven cancers), otherwise equal treatment leads to equal result [46]. Contrasting with the above-mentioned socioeconomic drivers of TNBC disparity among AA and EA women, the 2015 population based study from the SEER program revealed that the incidence and progression of hormone negative or triple negative breast cancer (TNBC) are independent of socioeconomic status [47]. A major caveat associated with population wide studies investigating racial disparity in AA and EA women is smaller sample sizes owing to lower participation rates and the fact that AA community is a minority (~12% of US population).

## 5. Molecular Pathways as Therapeutic Targets in Triple Negative Breast Cancer

Vascular endothelial growth factor (VEGF), androgen receptor (AR), mammalian target of rapamycin (mTOR), poly (ADP-ribose) polymerase (PARP), epidermal growth factor receptor (EGFR), histone deacetylase (HDAC), and Src oncogenic pathway are some of the major molecular pathways that are currently being targeted in TNBCs. The notion that VEGF inhibitors would prove to be effective against TNBC is based on the fact that TNBCs may require enhanced neoangiogenesis owing to their highly proliferative nature but no studies so far have found any TNBC specific effect of antiangiogenic agents [48]. Anti-VEGF monoclonal antibody, Bevacizumab’s clinical efficacy is currently being evaluated for metastatic TNBC. PARP inhibitors are under development as therapeutic agents for cancers with DNA repair defects like BRCA1/2 mutant TNBCs [49]. US Food and Drug Administration approved Olaparib, a PARP inhibitor, as a solitary-agent in December 2014 for advanced ovarian cancer patients with deleterious or suspected deleterious germ-line BRCA mutation who have had three earlier lines of chemotherapy. Many phase I and II trials have demonstrated antitumor activity of Olaparib in BRCA-mutated breast cancer patients [50,51,52,53]. To evaluate the efficacy and safety of Olaparib at two doses (one as maximum tolerated dose while the other as a lower pharmacodynamically active dose), a multi-center proof-of-concept phase II study was designed for BRCA1 and BRCA2 mutant advanced breast cancer [54]. Interestingly, 41% overall response rate (ORR) was noted in patients who were given 400 mg of Olaparib twice daily and 22% ORR was noted in patients who were administered with 100 mg of Olaparib twice daily. However, ORR was 54% and 25%, respectively, in triple negative breast cancer patients who were administered higher and lower doses of Olaparib, respectively. The OlympiAD trial had approximately 49% TNBC patients and the results demonstrated a significantly longer progression-free-survival (PFS) in the Olaparib group compared to standard therapy [55]. The safety, tolerability, and efficacy of Olaparib were evaluated in combination with paclitaxel in metastatic TNBC patients with ≤1 prior cytotoxic regimen and 37% of patients demonstrated partial response [56]. The combinational effects of veliparib and carboplatin in the neoadjuvant settings was investigated by Rugo et al. in two arms (paclitaxel monotherapy or paclitaxel-veliparib-carboplatin combinational therapy) of breast cancer patients [57]. The calculated PCR was observed to be 51% in paclitaxel-veliparib-carboplatin versus 26% in the control group [57]. The results from a phase II study of metastatic TNBC patients, O’Shaughnessy et al. showed that the inclusion of iniparib to gemcitabine and carboplatin enhanced the clinical benefit rate to 56% from 34% (*p* = 0.01) as well as the overall response rate to 52% from 32% (*p* = 0.02). Apart from this, iniparib inclusion also prolonged the median PFS from 3.6 months to 5.9 months with a median overall survival from 7.7 months to 12.3 months [58]. PARP inhibition seems to be a promising approach against TNBC (Table 1) but none of these trials focus on racial disparity. No race-specific information is available about the participants of these trials. The efficacy of these agents needs to be explored in multicenter clinical trials including AA as well as EA breast cancer patients [57].

Though the androgen receptor (AR) is present in normal as well as malignant breast tissue, its expression level varies according to the breast cancer subtype. AR is expressed in 10–15% of TNBCs [59,60]. Similarities exist between LAR and apocrine subtype and reports show that LAR subtype comprises breast cancer with apocrine histology [61,62]. Doane et al. identified a cell line exhibiting the molecular profile of LAR subtype [63], used it in preclinical models, validated its androgen reliant growth in estrogen free manner and successful inhibition using flutamide (AR antagonist). This study served as the first proof-of-principle study supporting androgen deprivation as a targeted therapy for LAR-TNBC [61,63]. Gucalp et al. conducted a multicenter Phase II study of bicalutamide at a dose of 150 mg per day in AR positive (12% of patients), and ER and PR negative metastatic breast cancer involving 26 patients (many of them were HER2 negative). A clinical benefit rate (CBR) of 19% for bicalutamide was revealed by this study with a median progression free survival (PFS) of 12 weeks [64]. Minor side effects included fatigue, hot flashes, limb edema, and transaminase elevation were observed [64]. Another multicenter phase II trial assessed the activity of next-generation anti-androgen enzalutamide in advanced AR positive TNBCs in two stages. In the first stage, 26 patients were given an oral dose of 160 mg per day of enzalutamide; assessed for the primary end point of the CBR at 16 weeks. The result demonstrated 42% CBR (95% confidence interval 24–62%) with one complete response and one partial response [65]. In the second stage, 165 patients were screened (75 patients had AR IHC ≤ 10% with more than one post baseline evaluation). Data revealed a CBR of 35% with a median progression free survival (PFS) of 14.7 weeks [66]. It would be worthwhile to mention an isolated clinical case of a heavily pretreated woman with TNBC and AR positivity who achieved a complete clinical response after 4 months of bicalutamide treatment [67]. An overview of main ongoing trials investigating AR targeting drugs is provided (Table 2) and it is interesting to note that none of these trials considered race-specific issues. With promising results, these new targeted therapies may also prove to be beneficial for AA TNBC but certainly requires careful assessment. Moving forward clinical studies focusing on the racial disparity in treatment response need to be conducted.

The fact that a large fraction of TNBC tumors overexpress EGFR puts forth EGFR as an attractive therapeutic target in TNBC. The combination of Cetuximab (monoclonal antibody) with Carboplatin revealed impressive effects on TNBC patients with a response rate of 17% [68]. In a randomized phase II trial, 173 metastatic TNBC patients pre-treated with only one previous line of chemotherapy for metastatic disease were given cisplatin either alone or in combination with cetuximab. Improvement in activity was noted in the combinational group with overall response rate (ORR) of 20% versus 10% with a progression free survival (PFS) of 3.7 versus 1.5 months [69]. Overexpression of Src tyrosine kinase is commonly observed in TNBC and has been associated with metastatic disease progression [70]. Preclinical evidence showed that dasatinib, an oral inhibitor of Src, c-kit and platelet derived growth factor receptor-β inhibits growth of TNBC cells. Moreover, the combination of dasatinib and cisplatin imparts synergistic effects in dasatinib-sensitive cells [70,71]. In a single arm phase I trial, dasatinib (70 mg twice daily) was given to metastatic TNBC patients pre-treated with anthracycline and a taxane showed a disease control rate of 9.3% with a 4.3% confirmed partial response rate along with a PFS of 8.3 weeks [72]. AKT and phosphatase and tensin homolog (PTEN) regulate PI3K signaling and mTOR is an effector node of PI3K cascade. PTEN loss is commonly observed in TNBC which leads to mTOR activation [73]. Presently, mTOR inhibitors are being assessed in TNBC or HER2 negative breast cancer patients [74]. The oral dose of 10 mg/day everolimus showed an overall response rate (ORR) of 12% in a phase II trial of first- or second-line treatment of metastatic breast cancer patients [74]. Aberrant Rb pathway is a significant feature of TNBC [75] and many druggable targets are present in this setting like Aurora kinase, polo-like kinase and Hsp90. In the TCGA data set, MYC is amplified in approximately 30% of basal-like breast cancers [75] which suggest CDK inhibition as a plausible targeting strategy in MYC amplified basal-like breast cancer [76]. Similarly, basal-like breast cancers are also driven by FOXM1 which also indicates that growth inducing cascades can be targeted for basal-like breast cancer [75]. TNBC exhibit resistance to taxanes and elevated MAPK/ERK pathway has been attributed to taxane resistance [77]. Hence, efficacy of MEK (a component of the MAPK/ERK pathway) inhibitor, cobimetinib, was tested in a randomized, phase II, double-blind, multicenter clinical trial in combination with paclitaxel in advanced TNBC patients [78]. Combination of cobimetinib and paclitaxel showed increased progression free survival. Several MEK inhibitors are being tested in clinical trials in combination with other targeted and chemotherapies (Table 3).

Platinum agents such as carboplatin, oxaliplatin and cisplatin function as DNA damaging compounds that cause DNA strand breaks resulting in apoptotic cell death hence they can be particularly effective in BRCA1/2 mutant TNBCs. Many studies have been investigating the effectiveness of platinum-based neoadjuvant chemotherapy in TNBC patients [79]. Meta-analysis of 2109 TNBC patients receiving either platinum-based (*n* = 1046) or platinum-free chemotherapy (*n* = 1063) observed a significant increase in pCR rate (15.1%) with platinum-based chemotherapy in the neoadjuvant setting [80]. Various immunotherapy strategies are being examined in TNBC including immune checkpoint blockade (Cytotoxic T-lymphocyte-associated actigen 4 (CTLA-4) inhibition, programmed death ligand-1/programmed cell death-1 PD-L1/PD-1 inhibition, CD47 checkpoint blockade), vaccines (DR5 DNA vaccination, dendritic cells (DC)-based vaccines etc.) and others with variable efficacy [81]. Combination therapy approaches encompassing standard therapy with immunotherapy are showing promise against TNBC. Evaluating a combination approach with Atezolizumab (targeting PD-L1) and Nanoparticle albumin-bound (nab)-paclitaxel, Schmid et al. observed prolonged progression-free survival [82]. Though it is still early to draw conclusive inferences with regard to immunotherapy in TNBC, completion of multiple ongoing clinical trials will provide evidence supporting the efficacy of combination regimens involving immunotherapy and standard chemotherapy.

Multiple new combinational or sequential therapies are currently under investigation in well-designed prospective clinical trials to counter TNBC based on their unique biology in this era of precision medicine. Even with considerable advancement in understanding the unique biology of TNBC in AA women owing to epidemiological, clinical and preclinical studies, very few clinical trials have undertaken the task to directly compare the efficacy of therapeutic agents in AA vs. EA TNBC patients; only one such trial is currently active (Table 4).

## 6. Cancer Stem Cells, Triple Negative Breast Cancer, and African-American Ancestry

Cancer stem cells (CSC), a rare population of cancer cells harboring the potential for self-renewal and differentiation, have been shown to exist in breast cancer. Some major characteristics of cancer stem cells are heterogeneity, asymmetric cell division, quiescence, and drug resistance enabling them to lead to cancer progression and relapse [83]. CSCs in breast cancer are generally characterized with a CD44+/CD24− and/or ALDH1+ (ALDH1A1+) population [84]. Nalwoga et al., observed that 48% breast cancer with African origin overexpressed ALDH1+ population [85] while only 19% and 30% breast tumors from non-African women in Michigan and France overexpressed ALDH1+ population [86]. Wnt signaling leads to progenitor cell renewal and is involved in embryologic development. A number of studies suggest the activation of Wnt signaling pathway in TNBC [87,88]. Microarray analysis demonstrated that *FZD7* (a major component of the Wnt signaling pathway) is upregulated in TNBC cell lines and tumor samples [89]. Increased LDL receptor related protein 6 (LRP6) upregulates Wnt signaling and leads to increased stemness in TNBC cells [90]. A study conducted by Getz et al., revealed that Wnt signaling pathway represents a major functional pathway in AA TNBC cohort [91] and genes associated with Wnt signaling like *TNC*, *Cav1*, *FOXO3A* and *TCF4* are significantly induced in AA TNBC [91]. Examination of *WNT10B* and its target gene, *HMGA2*, in TNBC cohorts showed that both *WNT10B* and *HMGA2* were upregulated in TNBCs-*WNT10B* (88% in AA and 73% in EA) and *HMGA2* (88% in AA and 80% in EA) [92]. Co-activation of Wnt and Hedgehog signaling in TNBC samples correlated with shorter recurrence and poor survival [93]. SMO, a major node of Hedgehog pathway directly activates MYCN which induces the expression of Cyclin-D1 and FOXM1 and FOXM1 is a transcription factor related to growth and progression of TNBC [94]. Nakshatri et al., studied the distribution of mammary stem cell phenotype in the healthy breast tissue samples from AA and EA women from the Susan G Komen Tissue Bank (Indianapolis, IN) and found that CD44+/CD24− and endothelial protein C-receptor (EPCR or APC receptor) +/epithelial cell adhesion molecule (EpCAM)− multipotent stem cells were significantly overexpressed in AA in comparison to EA [95]. Although the notion that an abundance of CSCs can potentially explain the inherently aggressive biology of TNBCs in AA women is gaining traction and is supported by multiple studies, additional population based studies are required to prove this concept.

## 7. Obesity, a Coconspirator in TNBC Disparity

According to the Center for Disease Control and Prevention (CDC), obesity is defined as a body mass index (BMI) ≥ 30 kg/m^2^ and these individuals are considered as metabolically unhealthy [96]. Waist/hip ratio (WHR) is also used to measure the abdominal obesity. According to the World Health Organization, a WHR of ≥ 0.85 is considered to be an increased risk for metabolic disorders [97]. A significant increase in prevalence of obesity (35.3% to 40.4%) was noted in United States between 2005 to 2014 and the incidence of severe obesity also increased from 7.4% to 9.9% [98,99]. The Carolina Breast Cancer Study advocates that high body mass index and high waist/hip ratio enhances the probability of basal-like TNBC in premenopausal AA women [17]. A retrospective study of 620 white patients with invasive breast cancer in West Virginia (one of the states where obesity rates are higher in the United States) showed higher occurrence of TNBC in younger patients (45%) compared to other breast cancer subtypes (27%) [100]. More women with TNBC were obese (50%) in comparison to women harboring other subtypes of breast cancer (36%) [100]. Stage 3 of the Carolina Breast Cancer Study included women from 44 North Carolina counties from 2008 to 2014. Here, the WHR was estimated across the highest and lowest groups (≥0.84 to <0.77) relative to TNBC basal-type subset. Among all, women with higher WHR had increased risk for developing basal-type subset of TNBC [101]. Both premenopausal and postmenopausal women with increased WHR had higher probability of developing basal-like TNBC subset in comparison to the women with lower WHR [101]. Importantly, premenopausal AA women revealed maximum prevalence of basal-like TNBC as well as the basal-like TNBC risk factors [101]. A case-control study examining the link between BMI and risk of breast cancer in AA and non-Hispanic EA, The Women’s Contraceptive and Reproductive Experiences (CARE), reported that premenopausal ER−/PR− breast cancer is inversely associated with the BMI of a woman at 18 years of age while the current BMI exhibits positive association with postmenopausal ER+/PR+ breast cancer [102]. To decipher the potential risk factors for early aggressive breast cancer among AA and non-Hispanic EA women, The Women’s Circle of Health Study was constituted as a multisite case-control study in New Jersey and New York. This study observed a significant reverse relation of high BMI with ER−/PR− breast cancer among postmenopausal women and an enhanced risk of premenopausal breast cancer was associated with increased WHR [103,104]. A collaboration of the four studies: the Carolina Breast Cancer Study [101], the Women’s Circle of Health Study [102], the Black Women’s Health Study [105], and the Multiethnic Cohort Study [106] constitute the African American Breast Cancer Epidemiology and Risk (AMBER) Consortium [107]. Overall goal of the AMBER Consortium was to examine the discrepancies among results observed within the individual studies regarding obesity (measured either by BMI and/or WHR) and TNBC connection. Focusing on both premenopausal and postmenopausal AA women, AMBER Consortium examined how obesity (both general and central obesity) might associate with breast cancer subtypes [107,108]. According to the AMBER Consortium, AA women are impacted by general and central obesity depending on their menopausal status and hormone receptor subtype [107]. Different studies showed varied trends associating obesity and breast cancer in AA women depending on the use of BMI or WHR. Higher recent BMI correlated with increased risk for ER+ breast cancer and decreased risk for TNBC in postmenopausal women. However, elevated BMI was linked to lower frequency of premenopausal ER+ cancer as well as all subtypes of postmenopausal cancer [107] in premenopausal women. Interestingly, studies using WHR as a measure of obesity showed that high WHR in postmenopausal women associated with all breast cancer subtypes whereas in premenopausal women high WHR was associated with an increased probability of premenopausal ER+ tumors but not others [107]. AMBER concludes that there may be multiple mechanisms associating obesity with TNBC and other subtypes of breast cancer in AA women [107].

**Figure 4:**
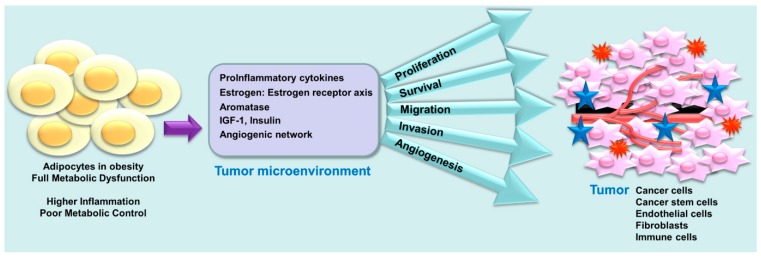
Adipocytes in obese state secrete proinflammatory cytokines in the tumor microenvironment and induce the major hallmarks of cancer development (proliferation, survival, migration, invasion, and angiognesis).

While most studies examining connection between obesity and disease prevalence use BMI, WHR or waist circumference, Capers et al., explored the importance of body shape in EA and AA women to predict disease associations [109]. In this study, 552 AA and EA women were recruited and body shapes, body fat distribution and body composition were recorded using digital photography, android-gynoid ratio (AGR) and dual-energy X-ray absorptiometry respectively. Interestingly, this study noted striking differences based on the race/ethnicity with AA women having higher mean BMI, mean AGR, mean total body fat, mean trunk fat, and mean leg fat in comparison to EA women. Also, apple body shape was more prevalent among AA women compared to EA women [109]. Obese AA and EA women also exhibit differences in distribution of adipose tissue, insulin resistance, and lipoprotein subclasses in intervention studies and interestingly only EA women showed decreased fasting insulin levels [110]. These clinical studies indicate that obesity in AA women might be different from obesity in EA women and may influence disease progression in a differential manner. Multiple studies have shown the negative impact of obesity on breast cancer progression including TNBC progression however no clinical trial has directly evaluated TNBC progression in obese AA and EA women. Obese people have increased circulating levels of insulin and inflammatory cytokines (IL-6, IL-8, TNF, and leptin) which can potentially induce STAT3, NFKB and EZH2 signaling [111] and may contribute to poor prognosis of AA TNBC (Figure 4). As obesity is common among AA women, obese state has been proposed as a major driver of aggressive TNBC biology in AA women.

## 8. Conclusions

**Figure 5:**
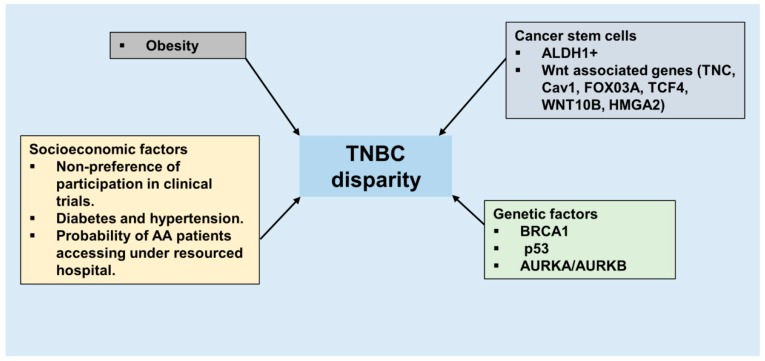
Biological and non-biological factors that contribute to TNBC disparity. Biological factors include obesity, cancer stem cell signaling pathways, markers, and genetic causes. Socioeconomic factors are major non-biological factors that play an important role in TNBC disparity.

Despite significant improvements in breast cancer diagnosis and therapeutics, TNBC remains a challenge owing to aggressive progression and lack of targeted therapies. In recent years, it has become evident that younger AA women are disproportionately affected by TNBC. Although many epidemiological and clinical studies have shown a higher prevalence of TNBC in women of African descent and put forth various genetic and environmental factors that may influence aggressive progression of TNBC in AA women, we still lack bigger studies focusing entirely on racial disparity in TNBC with emphasis on underprivileged AA women. Owing to the robust molecular classification, we now appreciate that TNBC subtype of breast cancer is heterogeneous in nature comprising of multiple sub-subtypes. In this era of personalized medicine, much effort has been dedicated to developing personalized therapeutic regimens based on molecular profile of breast cancer. To provide benefits of all the advancement in “personalized medicine” to AA TNBC patients in future, we need better understanding of the TNBC tumors arising in AA women. There are many unanswered queries such as deciphering the impact of genes vs. means in AA TNBC development; the molecular classification of AA TNBC tumors; understanding the prevalence of various TNBC subtypes in AA women, and elucidating the key molecular signaling pathway (Figure 5). It will be important to study various cancer stem cell signaling pathways (Wnt, Hedgehog and Notch) in AA TNBC as aberrant activation of cancer stem cells pathways may be the key node underlying the aggressive phenotype of AA TNBC and may explain disparities in AA TNBC progression. Obesity is known to disproportionately affect AA women and obese state has been associated with increased breast cancer incidence and progression hence a vital need exists to examine the role of obesity in driving TNBC in AA women. Higher TNBC incidence and an aggressive biology compounded with poor income, lower education, poor access to screening and standard treatment results in poor overall survival outcomes of TNBC in AA women. TNBC disparity in AA and EA women stems from a complex interaction of socioeconomic factors and biology. In conclusion, improving survival in AA TNBC cohort would require a transdisciplinary approach involving a cohesive interplay encompassing biology, genetics, socioeconomic factors, culture, and environment.

## Figures and Tables

**Table 1:** Ongoing trials investigating poly (ADP-ribose) polymerase (PARP) inhibitors in TNBC.

Phase	Clinical Trial	Treatment	ClinicalTrials.gov Identifier
Phase 1	A pilot study of Olaparib and Durvalumab in patients with metastatic triple negative breast cancer (TNBC)	Olaparib Durvalumab	NCT03544125
Phase 1	A phase I of Olaparib with Radiation Therapy in patients with inflammatory, loco-regional advanced or metastatic TNBC	Olaparib Radiation Therapy	NCT03109080
Phase 1	An open, non-randomized, multi-center Phase I study to access the safety and efficacy of Fluzoparib given in combination with Apatinib	Fluzoparib Apatinib	NCT03075462
Phase 1	A phase 1 study of PARP inhibitor Olaparib and HSP90 inhibitor AT13387	Olaparib Onalespib	NCT02898207
Phase 2	A phase II open-label, randomized study of PARP inhibition either alone or in combination with anti-PD-L1 Therapy	Atezolizumab Olaparib	NCT02849496
Phase 1 Phase 2	Phase 1/2 clinical study of Niraparib in combination with Pembrilizumab (MK-3475)	Niraparib Pembrolizumab	NCT02657889
Phase 2	A phase II clinical trial of the PARP inhibitor Talazoparib	Talazoparib Tosylate	NCT02401347

**Table 2:** Studies investigating androgen receptor (AR) inhibitors in TNBC.

Phase	Clinical Trial	Treatment	ClinicalTrials.gov Identifier
Phase 1/2	CYP17 Lyase and Androgen Receptor Inhibitor treatment with Seviteronel	Seviteronel	NCT02580448
Phase 1 Phase 2	A phase I/II, single arm, non-randomized study of Ribociclib (LEE011), a CDK 4/6 inhibitor, in combination with Bicalutamide, an androgen receptor (AR) inhibitor	Ribociclib Bicalutamide	NCT03090165
Phase 1 Phase 2	A phase Ib/II trial of Taselisib (GDC-0032), a PI3K inhibitor, in combination with Enzalutamide in patient with AR+veTNBC	Enzalutamide Taselisib	NCT02457910
Phase 2	A phase 2 open-label study to evaluate the efficacy and safety of VT-464, previous treatment with Enzalutamide	VT-464	NCT02130700

**Table 3:** Studies investigating mitogen-activated protein kinase kinase (MEK) inhibitors in TNBC.

Phase 1	Clinical Trial	Treatment	ClinicalTrials.gov Identifier
Phase 2	Neoadjuvant chemotherapy Docetaxel with or without Selumetinib in patients with TNBC	Selumetinib Doxorubicin Cyclophosphamide Docetaxel	NCT02685657
Early phase 1	Defining the TNBC kinome response to GSK1120212, MEK inhibitor	GSK1120212	NCT01467310
Phase 2	A single arm, phase II study of single agent Trametinib followed by Trametinib in combination with GSK21411795	Tramedtinib GSK21411795	NCT01964924
Phase 1	Safety, pharmacokinetics (PK) of AKT and MEK combination	GSK1120212 GSK21411795	NCT01138085
Phase 1	A study to investigate safety, pharmacokinetics and pharmacodynamics of BKM120 plus GSK1120212	BKM120 GSK1121212	NCT01155453
Phase 1	Safety, pharmacokinetics and pharmacodynamics of BKM120 plus MEK162	BEZ235 MEK162	NCT01337765
Phase 1	A phase Ib, Open-label, Multi-center, Dose-escalation, and Expansion Study of an Orally Administered Combination of BKM120 Plus MEK162	BKM120 MEK162	NCT01363232

**Table 4:** Study comparing response in patients of African ancestry (AA) and non-African ancestry.

Phase	Clinical Trial	Treatment	ClinicalTrials.gov Identifier
Phase 2	Study of CB-839 in combination w/Pacliatxel in patients of African Ancestry (AA) and Non-African (Non AA) Ancestry with Advanced TNBC	Glutaminase Inhibitor CB-839 in combination with Paclitaxel Cohort 1: AA, 3^rd^ line+ Cohort 2: AA, 1^st^ line Cohort 3: non-AA, 1^st^ line Cohort 4: non-AA, 1^st^ line	NCT03057600
